# Depletion of club cells attenuates bleomycin-induced lung injury and fibrosis in mice

**DOI:** 10.1186/s12950-017-0168-1

**Published:** 2017-09-18

**Authors:** Tetsuya Yokoyama, Toyoshi Yanagihara, Kunihiro Suzuki, Naoki Hamada, Kazuya Tsubouchi, Saiko Ogata-Suetsugu, Hironori Mikumo, Chika Ikeda-Harada, Takashige Maeyama, Kazuyoshi Kuwano, Yoichi Nakanishi

**Affiliations:** 1 Research Institute for Diseases of the Chest, Graduate School of Medical Sciences, Kyushu University, Fukuoka, Japan; 2 Division of Respiratory Diseases, Department of Internal Medicine, Jikei University School of Medicine, Tokyo, Japan

**Keywords:** Club cell, Lung injury, Lung fibrosis

## Abstract

**Background:**

The role of bronchiolar epithelial cells in the pathogenesis of pulmonary fibrosis has not been clarified. We previously demonstrated DNA damage in murine bronchioles in the early stages of bleomycin-induced pulmonary fibrosis that subsequently extended to alveolar cells at the advanced stages of the disease. Club cells are progenitor cells for bronchioles and are known to play protective roles against lung inflammation and damage. The aim of the present study was to elucidate the role of club cells in the development of pulmonary fibrosis.

**Methods:**

C57BL/6 J mice received naphthalene intraperitoneally on day −2 to deplete club cells and were given intratracheal bleomycin or a vehicle on day 0. Lung tissues were obtained on days 1, 7, and 14, and bronchoalveolar lavage was performed on day 14. Bronchiolar epithelial cells sampled by laser capture microdissection were analyzed by gene expression microarray analysis on day 14.

**Results:**

Club cell depletion induced by naphthalene protected mice from bleomycin-induced lung injury and fibrosis. Bleomycin-triggered bronchiolar TGF-β1 expression was reduced. Gene expression microarray analysis revealed that genes associated with inflammatory response and chemokine activity were downregulated in the bleomycin-injured bronchiolar epithelium with club cell injury compared to that in bronchiolar epithelium without cell injury.

**Conclusions:**

Club cells are involved in the development of lung injury and fibrosis.

## Background

Idiopathic pulmonary fibrosis is a representative disease of chronic fibrosing interstitial pneumonia of unknown etiology with the histopathological characteristics of usual interstitial pneumonia [1]. The prognosis of this disease is poor, and till date, effective treatments have not been sufficiently developed [1]. Although the pathogenesis of pulmonary fibrosis is not completely understood, it is thought that recurrent alveolar epithelial injury followed by aberrant repair eventually leads to this disease [2]. Therefore, it was previously believed that the predominantly affected region is the alveolar epithelial cells. However, our previous study demonstrated that apoptotic cells are found in the bronchiolar epithelial cells, as well as in some alveolar epithelial cells, around the bronchioles at 6 h after bleomycin instillation [3]*.* From day 1 to day 14, apoptotic bronchiolar epithelial cells disappeared, whereas apoptosis of alveolar epithelial cells was observed, and they distributed diffusely throughout the lung parenchyma [3].

Regional pulmonary stem cells are essential for tissue repair. Progenitor cell populations are basal cells in proximal airways and club cells, variant club cells, pulmonary neuroendocrine cells, and bronchioalveolar stem cells in distal airways [4, 5]. Progenitor cell dysfunction impairs regeneration and promotes lung injury [6]. Club cells are non-ciliated secretory epithelial cells that line bronchioles; these cells play progenitor roles and maintain homeostasis of bronchiolar walls [7–9]. Club cells have various functions, one of which is the augmentation of the inflammatory response by producing club cell secretory protein (CCSP) [10, 11]. Lipopolysaccharide (LPS) -induced pulmonary inflammation is augmented in chemically club cell-depleted mice and CCSP^−/−^ mice [11].

However, the role of club cells in the development of pulmonary fibrosis remains largely unclear. In the present study, we investigated the role of club cells in the development of pulmonary fibrosis by transiently depleting club cells using naphthalene, an aromatic hydrocarbon that is specifically cytotoxic to club cells [12].

## Methods

### Animal treatment

This experiment was approved by the Committee on Ethics regarding Animal Experiments of Kyushu University (reference number: A28–074-0) and was performed according to the guidelines of the American Physiological Society. Female C57BL/6 J mice (7 weeks old; Japan SLC, Shizuoka, Japan) were used in all experiments. Their initial body weights were 16 to 18 g. Naphthalene in corn oil (200 mg/kg; Wako Pure Chemical Industries, Osaka, Japan) was injected intraperitoneally on day −2. Controls were injected with corn oil only. The anesthetized mice were intratracheally administered 50 μl of 2 U/kg bleomycin hydrochloride (Nippon Kayaku, Tokyo, Japan) solution in saline on day 0. Controls were administered saline only. Mice were anesthetized and killed on days 1, 7, and 14.

### Histopathologic examination and immunohistochemistry

Histopathologic examination and pathological grading of lung injury were performed as previously described [13]. Sections were stained with Elastica van Gieson to assess collagen deposition. Immunohistochemistry was performed using the following method. Sections were deparaffinized with xylene and dehydrated in graded ethanol. Endogenous peroxidase activity was blocked with 0.3% hydrogen peroxide in methanol for 30 min at room temperature. Sections were autoclaved (121 °C, 5 min) to activate heat-mediated antigen retrieval. After blocking nonspecific proteins by incubating with normal serum, the sections were incubated with anti-CCSP antibodies (1:2000; Santa Cruz Biotechnology, Santa Cruz, CA) and anti-Trefoil factor 2 (TFF2) antibodies (1:150; Proteintech, Chicago, IL) at 4 °C overnight. Next, the sections were incubated with an immunoperoxidase polymer reagent, Histofine Simple Stain Mouse MAX PO (Nichirei Bioscience, Tokyo, Japan), for 30 min. Positive reactions were visualized with 3,3′-diaminobenzidine tetrahydrochloride (Nichirei Bioscience). The nuclei were lightly counterstained with hematoxylin. The representative immunostaining image for the control group was taken on day 14. Counting of the CCSP-positive cells in the bronchiolar epithelial cells was performed as previously described [14].

Collagen deposition was assessed by semi-quantitative morphological methods on paraffin-embedded samples stained with Elastica van Gieson. Twenty microscopic fields per animal were randomly registered by a digitizing camera attached to a light microscope with 200-fold magnification. On the digitized images, the area of the collagen fibers was measured using a fluorescence microscope (Biozero; KEYENCE, Osaka, Japan).

### Bronchoalveolar lavage

The bronchoalveolar lavage (BAL) method and analysis, as well as the measurement of the protein concentration, were performed as previously described [14].

### Enzyme-linked immunosorbent assay (ELISA)

The concentration of transforming growth factor-β1 (TGF-β1) in the BAL fluid supernatants was measured using commercial ELISA kits (R&D Systems, Minneapolis, MN) according to the manufacturer’s instructions. We performed all assays in duplicate, and the mean of two measurements was taken for each individual sample.

### Sampling with laser capture microdissection

Bronchiolar epithelial cells from lung tissues were sampled by laser capture microdissection using a Leica AS LMD (Leica Microsystems, Wetzlar, Germany) as previously described [14].

### Gene expression microarray and data analysis

Three mice were included in each group. Gene expression microarray and data analysis were performed as previously described [15]. Microarray data analysis was supported by Cell Innovator (Fukuoka, Japan). Total RNA was isolated from the cells extracted by laser capture microdissection using an RNeasy Micro Kit (Qiagen, Hilden, Germany) according to the manufacturer’s instructions. The RNA samples were quantified by an ND-1000 spectrophotometer (Thermo Fisher Scientific, Waltham, MA), and the quality was confirmed with an Agilent 2100 Bioanalyzer (Agilent Technologies, Santa Clara, CA). The complementary RNA was amplified, labeled, and hybridized to a SurePrint G3 Mouse GE 8 × 60 K Microarray (Agilent Technologies) according to the manufacturer’s instructions. All hybridized microarray slides were scanned using an Agilent scanner. Relative hybridization intensities and background hybridization values were calculated using Agilent Feature Extraction Software (version 9.5.1.1). The raw signal intensities and flags for each probe were calculated from the hybridization intensities (gProcessedSignal) and spot information (gIsSaturated, etc.) according to the procedures recommended by Agilent. (Flag criteria on GeneSpring Software. Absent (A): “Feature is not positive and significant” and “Feature is not above background.” Marginal (M): “Feature is not uniform,” “Feature is saturated,” and “Feature is a population outlier.” Present (P): Others.). The raw signal intensities of the two samples were log_2_-transformed and normalized using a quantile algorithm with the “preprocessCore” library package on Bioconductor software [16]. We selected probes that call “P” flag in both of the samples. To identify up or downregulated genes, we calculated the *Z*-scores and ratios (non-log-scaled fold-change) from the normalized signal intensities of each probe to compare the control and experiment samples. Then, we established criteria for the regulated genes: (upregulated genes) *Z*-score ≥ 2.0 and ratio ≥ 5-fold, (downregulated genes) *Z*-score ≤ −2.0 and ratio ≤ 0.2.The gene expression array data have been deposited in the Gene Expression Omnibus (accession number GSE94522).

The gene ontology analysis for differentially expressed genes was performed using DAVID with an all-mouse gene background and an enrichment score cutoff of more than 5.

### Statistical analysis

All data were statistically analyzed with *t*-tests between paired groups using JMP (version 9; SAS Institute, Cary, NC). *P* values less than 0.05 were considered statistically significant. Statistical analysis for the gene expression microarray was performed as described above.

## Results

### Naphthalene induces club cell injury

**Fig. 1:**
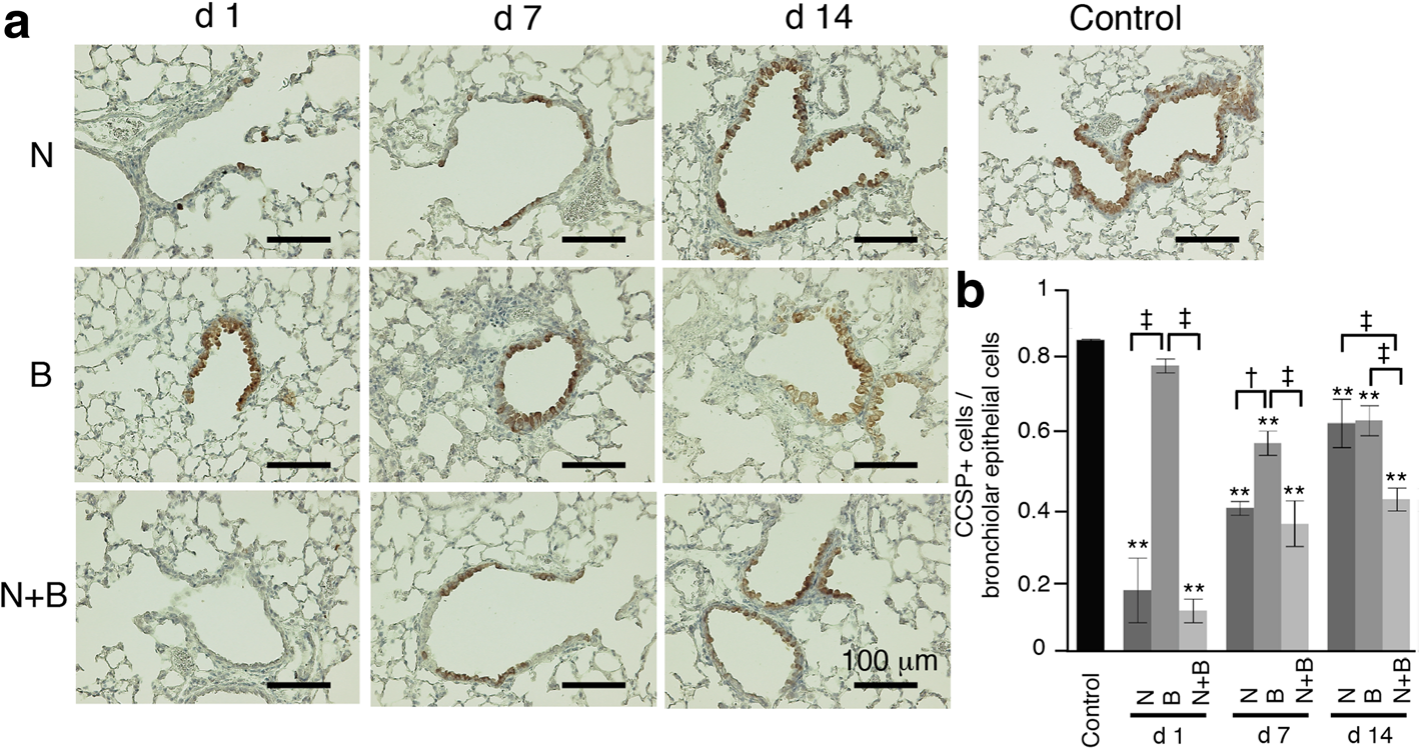
Assessment of naphthalene-induced club cell injury. C57BL/6 J mice were intraperitoneally injected with naphthalene or corn oil on day −2 and intratracheally administered bleomycin or vehicle on day 0. **a** Immunohistochemistry for CCSP in lung tissues was performed on days 1, 7, and 14, and representative images for the control, naphthalene alone, bleomycin alone, and bleomycin following naphthalene samples are shown. **b** Semi-quantified data of the ratio of the CCSP-positive cells to the bronchiolar epithelium. Data are presented as the mean ± SE of three mice. *P < 0.01 compared with control, ^†^ *P* < 0.05 and ^‡^ *P* < 0.01 compared between paired groups. N = naphthalene alone; B = bleomycin alone; N + B = bleomycin following naphthalene; CCSP = club cell secretory protein

We administered bleomycin intratracheally 2 days after the naphthalene injection. Immunohistochemistry for CCSP was performed to confirm the transient naphthalene-induced club cell depletion (Fig. 1). In the naphthalene-treated mice with or without bleomycin administration, most of the bronchiolar epithelial cells were negative for CCSP on day 1. A few CCSP-positive cells were detected on day 7, which subsequently almost recovered to the normal level on day 14 in the club cell-depleted mice. The mice treated with bleomycin after club cell depletion showed a similar tendency of CCSP alteration with naphthalene alone. However, the subsequent recovery of the CCSP-positive cells was delayed compared to treatment with naphthalene alone.

### Depletion of club cells attenuates bleomycin-induced lung injury and fibrosis

**Fig. 2:**
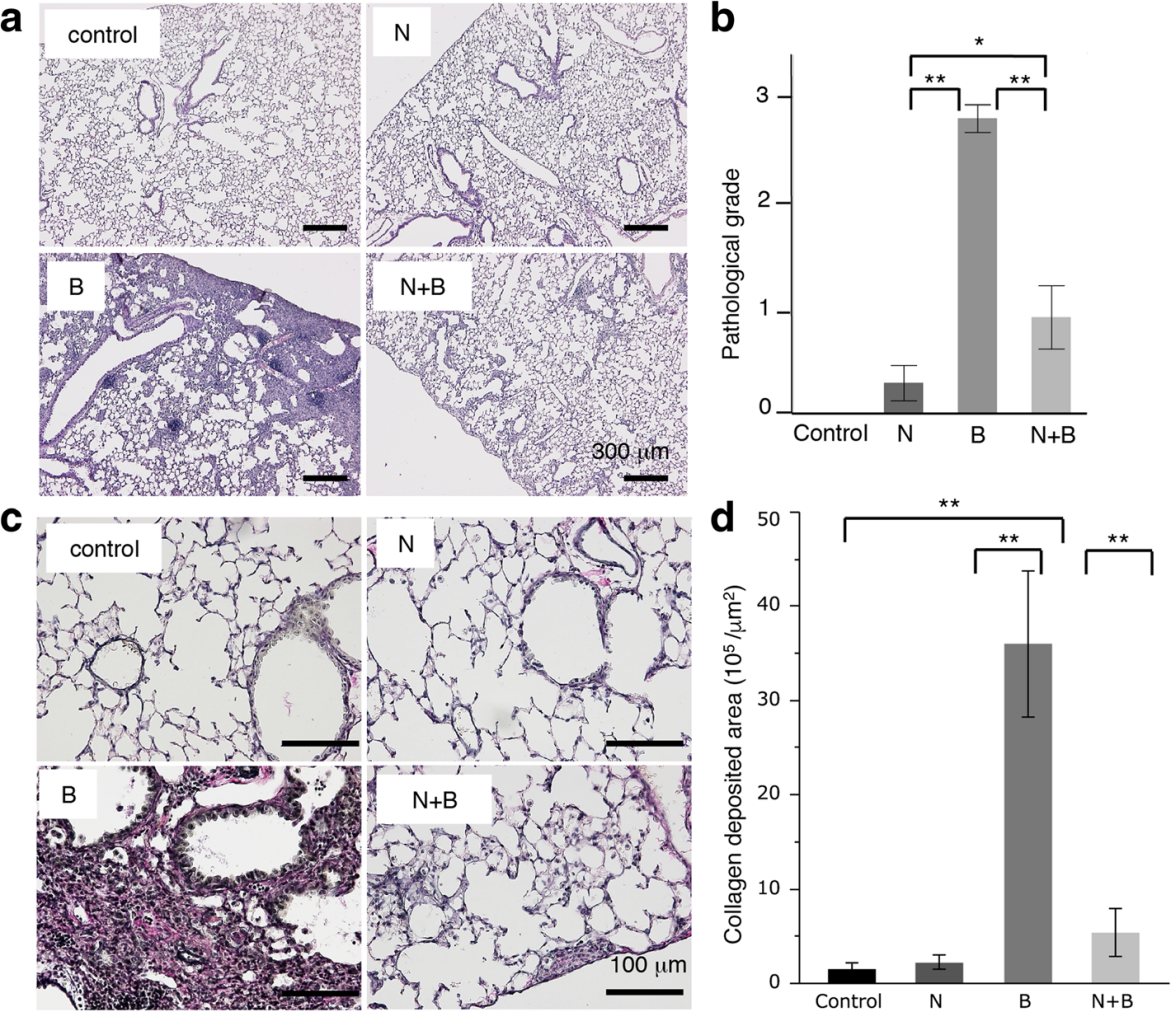
Depletion of club cells attenuated bleomycin-induced lung injury and fibrosis. **a** Hematoxylin and eosin staining of lung tissues from mice treated with control, naphthalene alone, bleomycin alone, and bleomycin following naphthalene. **b** Quantitative results of the pathological grade of lung injury. **c** Elastica van Gieson staining of lung tissues from the mice treated with control, naphthalene alone, bleomycin alone, and bleomycin following naphthalene on day 14. **d** Quantitative results of collagen deposition in the lung tissues. Data are presented as the mean ± SE of three mice in each group. *P < 0.05 and **P < 0.01. N = naphthalene alone; B = bleomycin alone; N + B = bleomycin following naphthalene

To elucidate the involvement of club cells in the pathogenesis of lung injury and fibrosis, we performed histological analysis and BAL fluid analysis on day 14. Hematoxylin and eosin staining of the lung tissues revealed that intratracheal bleomycin instillation caused infiltration of the inflammatory cells into the lung interstitium and thickening of the alveolar septa. However, club cell depletion significantly attenuated bleomycin-induced infiltration of the inflammatory cells and thickening of the alveolar septa (Fig. 2a, b). Elastica van Gieson staining revealed that club cell depletion also attenuated bleomycin-induced collagen deposition in the lung (Fig. 2c, d).

**Fig. 3:**
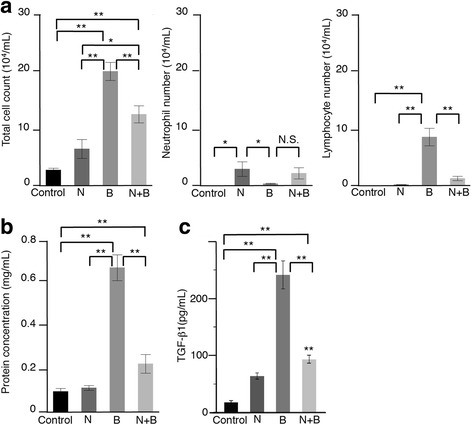
Depletion of club cells suppressed the total cell count, protein concentration, and TGF-β1 production in the bronchoalveolar lavage fluid. **a** The cell count (white blood cells, neutrophils, and lymphocytes), **b** protein concentration, and **c** TGF-β1 levels in the BAL fluid on day 14. Data are presented as the mean ± SE of 7–15 mice in each group. *P < 0.05 and **P < 0.01. N = naphthalene alone; B = bleomycin alone; N + B = bleomycin following naphthalene

Club cell depletion also reduced the total cell count, lymphocyte count, and protein concentration in the BAL fluid (Fig. 3a, b), which is consistent with the histological results. We believe that the higher neutrophil count in the club cell-depleted mice is due to the naphthalene induction of neutrophil migration to the airways, as previously described [14].

**Fig. 4:**
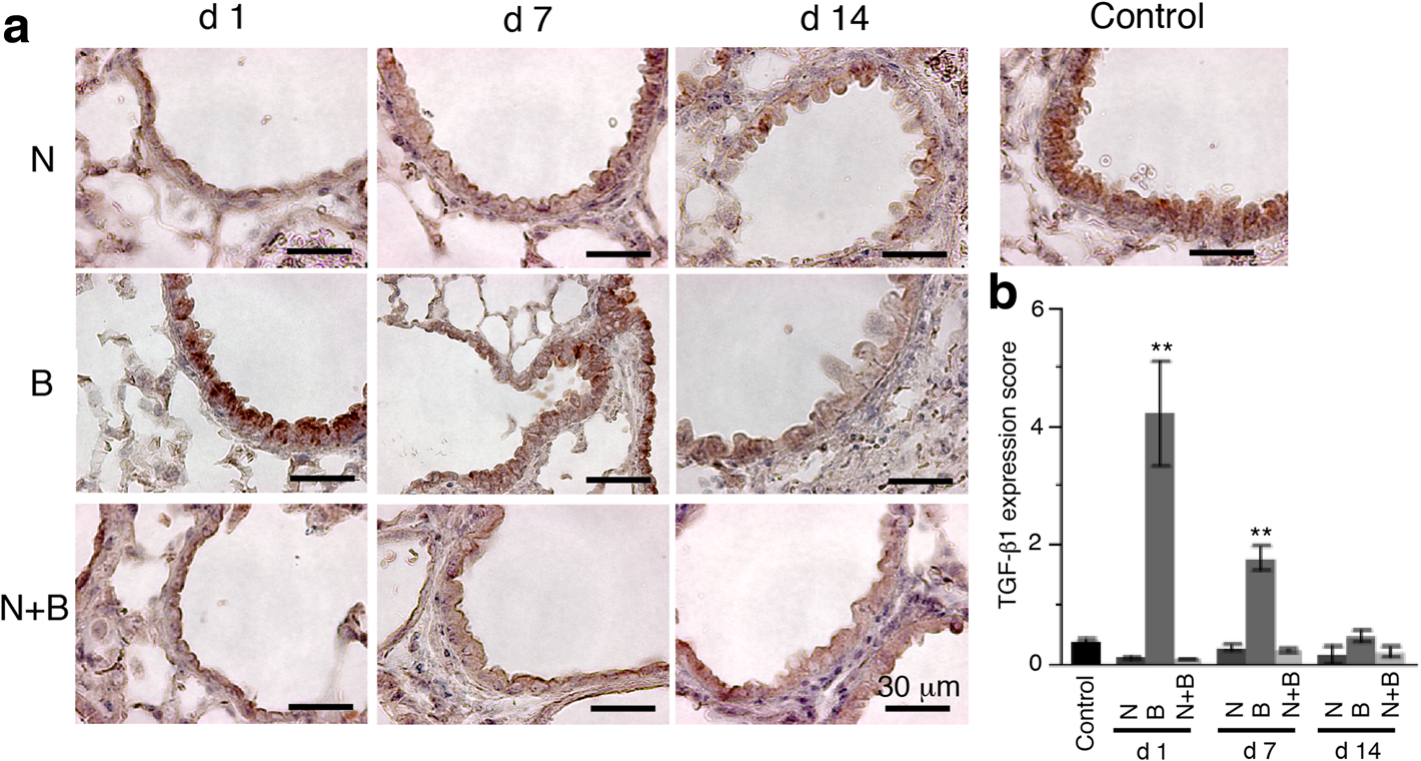
Upregulated expression of TGF-β1 protein in bronchiolar epithelial cells by intratracheal bleomycin instillation was inhibited by naphthalene-induced club cell injury. **a** Immunohistochemistry for TGF − β1 of lung tissues on days 1, 7, and 14. Representative image of control, naphthalene alone, bleomycin alone, and bleomycin following naphthalene were shown. **b** Semi-quantitative analysis of TGF-β1 expression. The intensity of immunostaining (0 = negative, 1–3 = weak, 4–6 = intense) was assessed in 20 bronchioles per each sample. Data are represented as mean ± SE of three mice each group. **P < 0.01 compared with all other groups. N = naphthalene alone; B = bleomycin alone; N + B = bleomycin following naphthalene

TGF-β1 plays central roles in the fibrogenesis, and its expression is upregulated in activated bronchiolar and alveolar type II cells and macrophages in idiopathic pulmonary fibrosis [17]. Club cell depletion reduced bleomycin-induced TGF-β1 in the BAL fluid (Fig. 3c). Similar results were obtained from the immunohistostaining of TGF-β1, especially in the early phase (Fig. 4). These results suggest that club cells play an important role in bleomycin-induced pulmonary fibrosis.

### Gene expression microarray analysis of bronchiolar epithelial cells

**Fig. 5:**
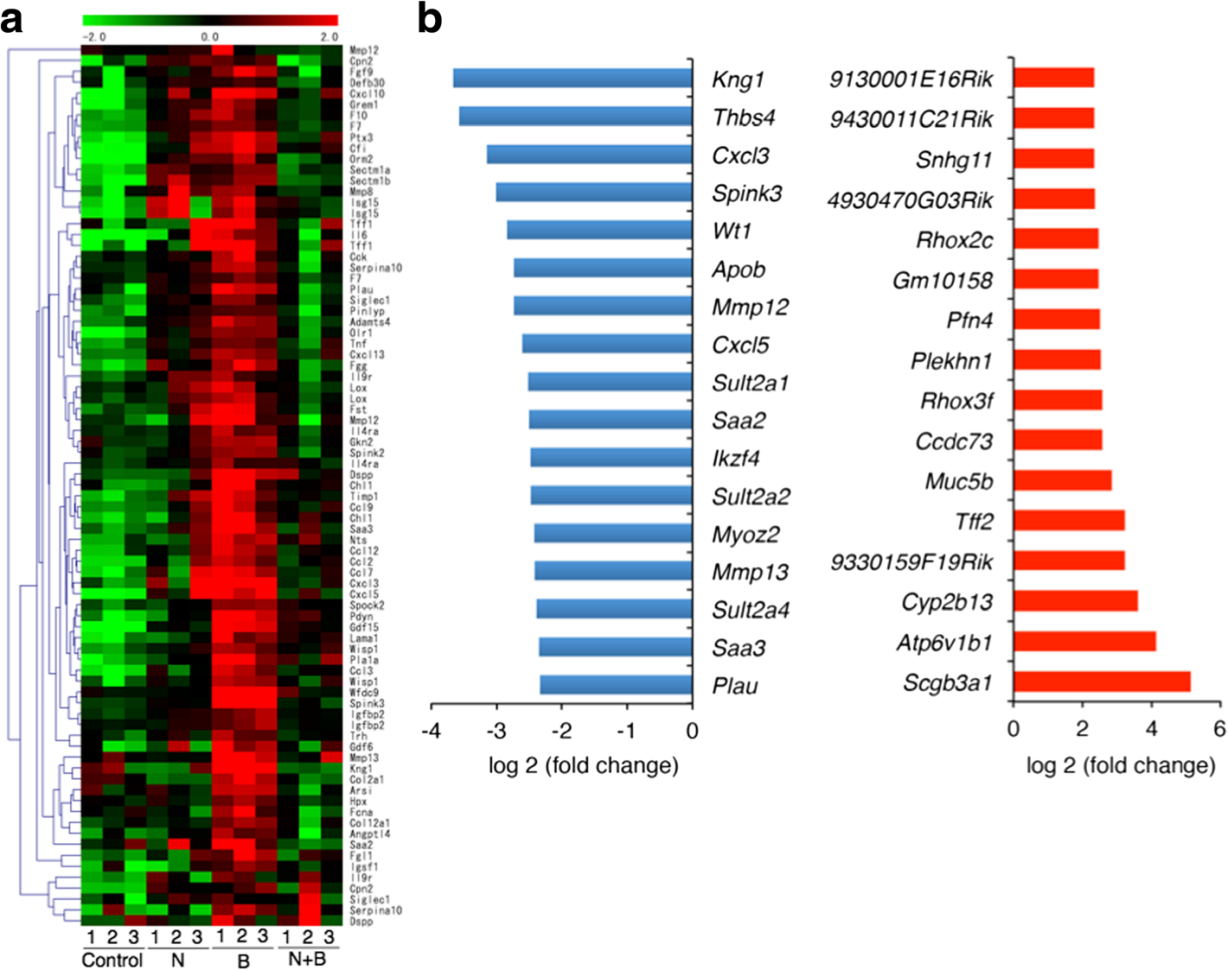
Gene expression analysis of the bronchiolar epithelium from bleomycin-treated mice with naphthalene-induced club cell injury compared to that without cell injury on day 14. **a** Heat map of the expression levels of selected genes in different clusters (red, upregulated; green, downregulated). **b** Genes downregulated and upregulated by bleomycin in the bronchiolar epithelium with club cell injury compared to those without injury on day 14. N = naphthalene alone; B = bleomycin alone; N + B = bleomycin following naphthalene

**Fig. 6:**
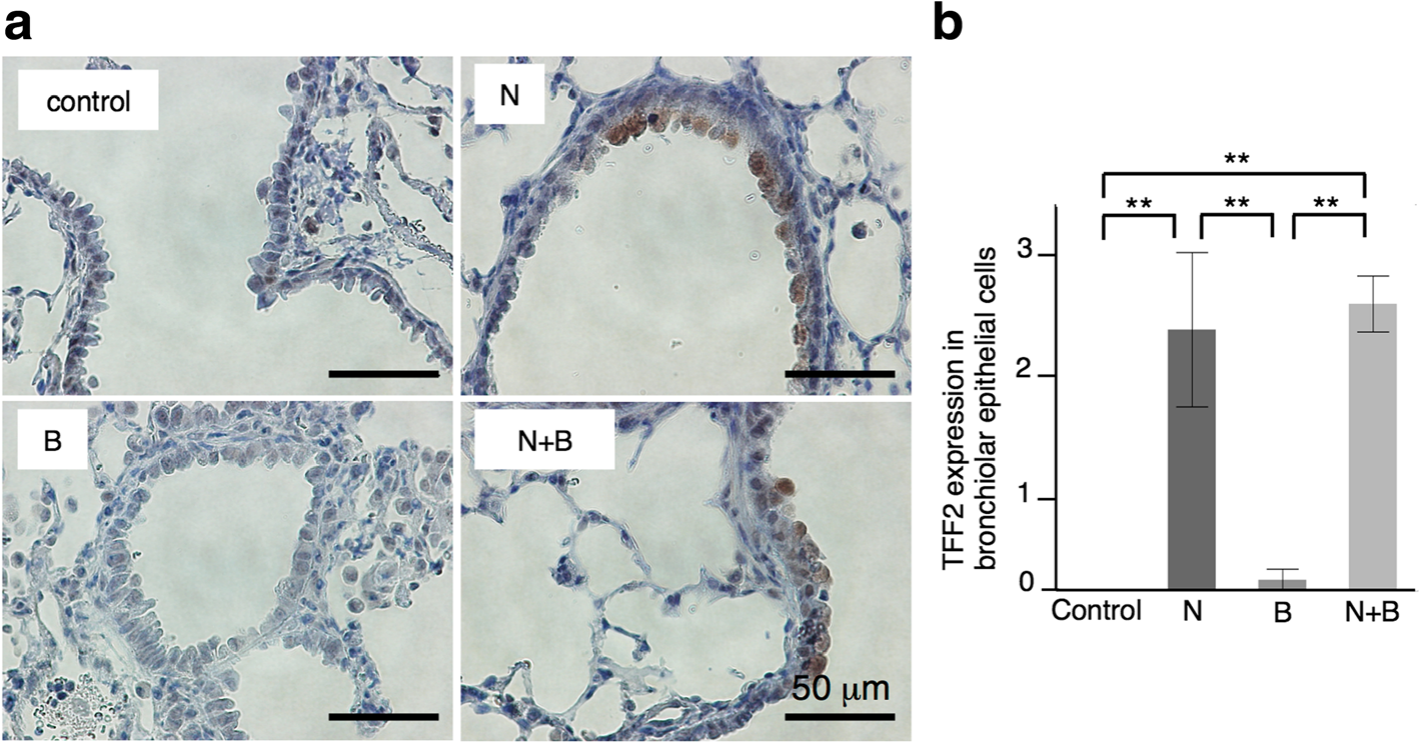
TFF2 expression in the bronchiolar epithelial cells. **a** Immunohistochemistry for TFF2 of lung tissues on day 14. **b** Semi-quantitative analysis of TFF2 expression. The intensity of immunostaining (0 = negative, 1 = weak, 2 = intense) and the percentage of positive cells (1 = <50%, 2 = 50–75%, 3 = >75%) were assessed in 20 bronchioles per each sample. The scores of each sample were multiplied to give a final score of 0 to 6. **P < 0.01. TFF2, trefoil factor 2. N = naphthalene alone; B = bleomycin alone; N + B = bleomycin following naphthalene

To explore the underlying mechanism why naphthalene-induced club cell injury behaved protectively against bleomycin-induced lung injury, we next performed gene expression microarray analysis on bronchiolar epithelial cells selectively obtained from the lung tissues by laser capture microdissection on day 14. The gene expression patterns for the bleomycin-injected mice with and without club cell depletion are shown in Fig. 5a. The bleomycin-injected mice with club cell depletion showed 17 markedly downregulated genes and 16 markedly upregulated genes compared to those without club cell depletion (Fig. 5b). To confirm this result, we performed immunostaining for one of the upregulated genes, *Tff2* (Fig. 6).

Gene ontology analysis showed that the biological process terms “response to wounding,” “inflammatory response,” and “defense response” were significantly downregulated in bleomycin-injected mice with club cell depletion compared with those without. Further, the molecular function terms “chemokine activity” and “chemokine receptor binding” were also significantly downregulated (Table 1). On the contrary, “cytokine–cytokine receptor interaction,” “extracellular matrix–receptor interaction,” and “complement and coagulation cascades” were significantly upregulated (Table 2). These results suggest that excessive wound response and inflammation response in club cells worsen pulmonary fibrosis.

**Table 1:** Gene ontology analysis for downregulated genes in the bronchiolar epithelium of bleomycin-treated mice with naphthalene-induced club cell injury compared to that without cell injury on day 14

Gene ontology term	Frequency (%)	*P* value	FDR (%)
Biological process			
Response to wounding	5.48	1.34 × 10^−15^	2.33 × 10^−12^
Inflammatory response	3.75	1.41 × 10^−11^	2.47 × 10^−8^
Defense response	4.90	3.28 × 10^−8^	5.75 × 10^−5^
Molecular function			
Chemokine activity	1.25	1.68 × 10^−7^	2.52 × 10^−4^
Chemokine receptor binding	1.25	2.32 × 10^−7^	3.48 × 10^−4^
Cytokine activity	2.59	8.90 × 10^−7^	0.001
Biological process			
Chemotaxis	2.02	2.52 × 10^−7^	4.42 × 10^−4^
Taxis	2.02	2.52 × 10^−7^	4.42 × 10^−4^
Locomotory behavior	2.69	3.99 × 10^−5^	0.070
Molecular function			
Serine-type endopeptidase inhibitor activity	1.92	3.55 × 10^−6^	0.005
Endopeptidase inhibitor activity	2.31	4.57 × 10^−6^	0.007
Peptidase inhibitor activity	2.40	6.48 × 10^−6^	0.010
Enzyme inhibitor activity	2.88	1.07 × 10^−5^	0.016

**Table 2:** Gene ontology analysis for upregulated genes in the bronchiolar epithelium of bleomycin-treated mice with naphthalene-induced club cell injury compared to that without cell injury on day 14

Pathway	Frequency (%)	*P* value	FDR (%)
Cytokine–cytokine receptor interaction	3.53	2.55 × 10^−15^	3.12 × 10^−12^
Extracellular matrix–receptor interaction	1.25	3.95 × 10^−6^	0.005
Complement and coagulation cascades	1.14	9.85 × 10^−6^	0.012

*FDR* false discovery rate

## Discussion

The aim of the present study was to elucidate the role of bronchiolar epithelial cells in the fibrotic milieu. During the planning phase, we speculated that depletion of club cells exacerbates bleomycin-induced pulmonary fibrosis. However, our results show that club cell-depleted mice are unexpectedly resistant to bleomycin-induced lung injury and fibrosis.

We demonstrated the involvement of bronchiolar epithelial cells in the development of bleomycin-induced lung injury and fibrosis. Naphthalene-induced club cell injury suppressed bleomycin-triggered TGF-β1 expression in the bronchiolar epithelium (Fig. 4). The expression was strongly inhibited on day 1. Our previous study has demonstrated that intratracheal bleomycin instillation induced apoptotic cells and proinflammatory mediator high mobility group box 1 expression in the bronchiolar epithelial cells [3, 13]. In the model of bleomycin-induced lung injury, bronchiolar epithelial cells were first affected and then expressed proinflammatory and profibrotic mediators. A recent report has demonstrated that tumor necrosis factor (TNF) superfamily protein 14, also known as LIGHT upregulated thymic stromal lymphoprotein (TSLP), with binding to its receptors on human and murine bronchiolar epithelial cells [18]. The inhibition of TSLP production by blocking LIGHT binding to its receptors attenuated bleomycin-induced pulmonary fibrosis [18]. These findings indicate that several mediators derived from bronchiolar epithelial cells play a crucial role in bleomycin-induced lung injury and fibrosis.

A number of genes expressions were markedly altered in the bleomycin-injured bronchiolar epithelial cells with club cell injury compared to those without injury. The downregulated genes contained some mediators related to pulmonary fibrosis, such as *Mmp12* and *Mmp13* (Fig. 4b). Matrix metalloproteinases (MMPs) degrade the various components of the extracellular matrix proteins and are involved in tissue remodeling and maintenance [19]. MMP-12 reportedly has fibrotic activity in Fas-induced pulmonary fibrosis [20], and MMP-13 has shown similar activity in radiation-induced pulmonary fibrosis [21]. Thus, the downregulation of MMPs may be involved in the improvement of lung fibrosis and injury.

The most upregulated gene *Scgb3a1* codes for the Scgb3a1 protein, which is a member of the secretoglobin family that includes CCSP and Scgb3a2; *Scgb3a1* is predominantly expressed in the epithelium of mammalian airways [22]. Exogenous Scgb3a2 administration has been shown to suppress bleomycin-induced pulmonary fibrosis via TGF-β signaling downregulation [23]. *Tff2* is also upregulated; this gene codes for TFF2, which is secreted as peptides from many mucin-producing cells. *Tff2* is associated with injury and repair in the gastrointestinal tract by various functions, including the anti-apoptotic effect and migration promotion [24]. The exogenous TFF2 treatment has been shown to reverse airway remodeling in mice model of allergic airway disease [25]. Unlike club cells, regenerated bronchiolar epithelial cells have anti-inflammatory, anti-fibrotic characters.

The alterations in gene expressions at bronchiolar epithelium suggest that club cells act as a conductor of pulmonary fibrosis. Intratracheal bleomycin instillation damages the bronchioles first [3], which induces the production of profibrotic and proinflammatory mediators in the club cells. Thus, excessive wound response and inflammation response in club cells worsen pulmonary fibrosis.

It was unexpected that the depletion of club cells attenuated bleomycin-induced lung injury. Because, previous reports showed that the depletion of club cells or CCSP deficiency augmented LPS-induced lung inflammation and induced alveolar dysfunction [6, 11]. In CCSP^−/−^ mice, LPS increased TNF-α signaling via toll-like receptor 4 (TLR4) on macrophages [11]. Bleomycin-induced pulmonary fibrosis was augmented in TLR4^−/−^ mice [26]. The result indicates that an alteration of TLR4 expression by naphthalene administration is partly responsible for the suppression of bleomycin-induced lung injury. Alveolar inflammation and depletion of alveolar type II cells were found in transgenic mice that were not able to regenerate bronchiolar epithelium after club cell injury, but not in naphthalene-induced club cell injury [6]. It suggests that airway regenerative capacity is involved in the maintenance of alveolar function and homeostasis. Thus, we speculate that alveolar stability mediated by rapid airway reconstruction after club cell injury results in the suppression of bleomycin-induced lung injury.

There were two limitations in the present study. First, microarray analysis was performed on day 14, when the CCSP-positive cells had mostly been repaired in the naphthalene-treated mice. Therefore, the gene expression profile on day 14 cannot determine whether active or inactive genes are responsible for the lung injury. Second, because we used naphthalene to transiently deplete club cells, we could not rule out the effect of the naphthalene on the alveolar epithelial cells. A genetically CCSP-deficient (CCSP^−/−^) mouse model should be used in further research to assess the effect oh the naphthalene.

## Conclusions

In conclusion, we demonstrated that naphthalene-induced club cell depletion protects mice from bleomycin-induced lung injury and fibrosis. Although the pathogenesis of pulmonary fibrosis remains to be fully elucidated, we have demonstrated the involvement of club cells in this disease.

## Abbreviations

BAL: bronchoalveolar lavage
CCSP: club cell secretory protein
ELISA: enzyme-linked immunosorbent assay
LPS: lipopolysaccharide
MMP: matrix metalloproteinases
TFF2: trefoil factor 2
TGF-β1: transforming growth factor-β1
TLR4: toll-like receptor 4
TNF: tumor necrosis factor
TSLP: thymic stromal lymphoprotein

## References

[1] Raghu G, Collard HR, Egan JJ, Martinez FJ, Behr J, Brown KK (2011). An official ATS/ERS/JRS/ALAT statement: idiopathic pulmonary fibrosis: evidence-based guidelines for diagnosis and management. Am J Respir Crit Care Med.

[2] Selman M, King TE, Pardo A (2001). American thoracic society, European respiratory society, American college of chest physicians. Idiopathic pulmonary fibrosis: prevailing and evolving hypotheses about its pathogenesis and implications for therapy. Ann Intern Med.

[3] Hagimoto N, Kuwano K, Kawasaki M, Yoshimi M, Kaneko Y, Kunitake R (1999). Induction of interleukin-8 secretion and apoptosis in bronchiolar epithelial cells by Fas ligation. Am J Respir Cell Mol Biol.

[4] Coraux C, Roux J, Jolly T, Birembaut P (2008). Epithelial cell-extracellular matrix interactions and stem cells in airway epithelial regeneration. Proc Am Thorac Soc.

[5] Kim CF, Jackson EL, Woolfenden AE, Lawrence S, Babar I, Vogel S (2005). Identification of bronchioalveolar stem cells in normal lung and lung cancer. Cell.

[6] Reynolds SD, Giangreco A, Hong KU, McGrath KE, Ortiz LA, Stripp BR (2004). Airway injury in lung disease pathophysiology: selective depletion of airway stem and progenitor cell pools potentiates lung inflammation and alveolar dysfunction. Am J Physiol Lung Cell Mol Physiol.

[7] Singh G, Katyal SL (1997). Clara cells and Clara cell 10 kD protein (CC10). Am J Respir Cell Mol Biol.

[8] Stripp BR, Reynolds SD (2008). Maintenance and repair of the bronchiolar epithelium. Proc Am Thorac Soc.

[9] Reynolds SD, Malkinson AM (2010). Clara cell: progenitor for the bronchiolar epithelium. Int J Biochem Cell Biol.

[10] Hermans C, Bernard A (1999). Lung epithelium-specific proteins: characteristics and potential applications as markers. Am J Respir Crit Care Med.

[11] Snyder JC, Reynolds SD, Hollingsworth JW, Li Z, Kaminski N, Stripp BR (2010). Clara cells attenuate the inflammatory response through regulation of macrophage behavior. Am J Respir Cell Mol Biol.

[12] Van Winkle LS, Buckpitt AR, Nishio SJ, Isaac JM, Plopper CG (1995). Cellular response in naphthalene-induced Clara cell injury and bronchiolar epithelial repair in mice. Am J Phys.

[13] Hamada N, Maeyama T, Kawaguchi T, Yoshimi M, Fukumoto J, Yamada M (2008). The role of high mobility group box1 in pulmonary fibrosis. Am J Respir Cell Mol Biol.

[14] Harada C, Kawaguchi T, Ogata-Suetsugu S, Yamada M, Hamada N, Maeyama T (2011). EGFR tyrosine kinase inhibition worsens acute lung injury in mice with repairing airway epithelium. Am J Respir Crit Care Med.

[15] Sekiya S, Suzuki A (2011). Direct conversion of mouse fibroblasts to hepatocyte-like cells by defined factors. Nature.

[16] Huang DW, Sherman BT, Lempicki RA (2009). Systematic and integrative analysis of large gene lists using DAVID bioinformatics resources. Nat Protoc.

[17] Khalil N, O'Connor RN, Unruh HW, Warren PW, Flanders KC, Kemp A (1991). Increased production and immunohistochemical localization of transforming growth factor-beta in idiopathic pulmonary fibrosis. Am J Respir Cell Mol Biol.

[18] Herro R, Antunes DSR, Aguilera AR, Tamada K, Croft M (2015). Tumor necrosis factor superfamily 14 (LIGHT) controls thymic stromal lymphoprotein to drive pulmonary fibrosis. J Allergy Clin Immunol.

[19] Löffek S, Schilling O, Franzke C-W (2011). Series “matrix metalloproteinases in lung health and disease”: biological role of matrix metalloproteinases: a critical balance. Eur Respir J.

[20] Matute-Bello G, Wurfel MM, Lee JS, Park DR, Frevert CW, Madtes DK (2007). Essential role of MMP-12 in Fas-induced lung fibrosis. Am J Respir Cell Mol Biol.

[21] Flechsig P, Hartenstein B, Teurich S, Dadrich M, Hauser K, Abdollahi A (2010). Loss of matrix metalloproteinase-13 attenuates murine radiation-induced pulmonary fibrosis. Int J Radiat Oncol Biol Phys.

[22] Reynolds SD, Reynolds PR, Pryhuber GS, Finder JD, Stripp BR (2002). Secretoglobins SCGB3A1 and SCGB3A2 define secretory cell subsets in mouse and human airways. Am J Respir Crit Care Med.

[23] Kurotani R, Okumura S, Matsubara T, Yokoyama U, Buckley JR, Tomita T (2011). Secretoglobin 3A2 suppresses bleomycin-induced pulmonary fibrosis by transforming growth factor beta signaling down-regulation. J Biol Chem.

[24] Kjellev S (2009). The trefoil factor family - small peptides with multiple functionalities. Cell Mol Life Sci.

[25] Greeley MA, Van Winkle LS, Edwards PC, Plopper CG (2010). Airway trefoil factor expression during naphthalene injury and repair. Toxicol Sci.

[26] Yang HZ, Wang JP, Mi S, Liu HZ, Cui B, Yan HM (2012). TLR4 activity is required in the resolution of pulmonary inflammation and fibrosis after acute and chronic lung injury. Am J Pathol.

